# A Low-Cost Chamber Prototype for Automatic Thermal Analysis of MEMS IMU Sensors in Tilt Measurements Perspective

**DOI:** 10.3390/s19122705

**Published:** 2019-06-16

**Authors:** Giuseppe Ruzza, Luigi Guerriero, Paola Revellino, Francesco M. Guadagno

**Affiliations:** Department of Science and Technology, University of Sannio, 82100 Benevento, Italy; giuseppe.ruzza@unisannio.it (G.R.); paola.revellino@unisannio.it (P.R.); guadagno@unisannio.it (F.M.G.)

**Keywords:** Micro Electro-Mechanical Systems (MEMS), accelerometer, thermal chamber, Arduino^®^, Peltier, tilt

## Abstract

In this work, a low-cost, open-source and replicable system prototype for thermal analysis of low-cost Micro Electro-Mechanical Systems (MEMS) Inertial Measurement Unit (IMU) sensors in tilt measurement perspective is presented and tested. The system is formed of a 3D printed frame, a thermal cell consisting in a Peltier element mounted over a heat sink, and a control and power system. The frame is designed to allow the independent biaxial tilting of the thermal cell through two servomotors. The control board is formed by an Arduino^®^ and a self-made board including a power drive for controlling the thermal unit and servomotors. We tested the chamber analyzing the behavior of multiple MEMS IMU onboard accelerometers suitable for measuring tilt. Our results underline the variability of the thermal behavior of the sensors, also for different sensor boards of the same model, and consequently the need for the adoption of a thermal compensation strategy based on thermal analysis results. These data suggesting the need for the analysis of the thermal behavior of MEMS-based sensors, indicate the potential of our system in making low-cost sensors suitable in medium-to-high precision monitoring applications.

## 1. Introduction

MEMS (Micro Electro Mechanical Systems) sensors such as accelerometers and gyroscope, have the potential to be used in a number of monitoring tools. Tunnels lining, bridges and buildings health, landslide displacement monitoring, and early warning are only few examples of possible applications. The authors of the study conducted in Reference [1] used triaxial MEMS accelerometers for the analysis of sway movement of trees in response to external forcing. Feng et al. [2] tested the potential of using smartphone accelerometers [3] for measuring the structural vibrations in buildings. The authors of the study in Reference [4] derived ground subsidence caused by the construction of the South Hongmei Road in Shangai with a self-developed, wireless, monitoring system based on MEMS. Milne et al. [5] applied MEMS sensors as a monitoring system to analyze railway response to passing trains and assess change in track health. Huang et al. [6] used MEMS accelerometer for monitoring and measuring the tilting deformation of a tunnel segment. The authors of the study in Reference [7] used MEMS tilt sensors, associated with volumetric water content sensors, to develop an early warning system for landslide applications. The authors of the studies in References [8,9,10] used the MEMS accelerometer sensor for application in SHM (Structural Health Monitoring). The authors of the study in [11] developed a thermal convection-based MEMS sensor that can be applied to both acceleration and inclination measurements.

Traditional systems used in precise monitoring of deformations for environmental and engineering applications are often based on electromechanical sensors. These sensors have the advantage of high accuracy and resolution and high measuring stability, but their cost is often prohibitive for most of the applications. For this reason, the monitoring systems are composed only of a few stations. Other disadvantages are their high-power consumption and large size. In this context, recently developed low-cost IMU (Inertial Measurement Unit) MEMS provide an opportunity to overcome this drawback [12]. In fact, they are being increasingly used in monitoring applications also in association with open-source controlling platforms such as Arduino^®^ [13,14,15,16]. Despite advantages over traditional high precision electromechanical sensor such as smaller size and power consumption and sufficient resolution for many monitoring problems, IMU MEMS sensors have the disadvantage to be very sensible to temperature variation [17,18]. This makes IMU MEMS not readily suitable for mid-to-high precision monitoring applications in uncontrolled environment.

On this basis, and following the work of Ruzza et al. [19] that indicated the possibility of mitigating thermal drift of MEMS IMU onboard accelerometers on the basis of data derived by a thermal chamber-based analysis, we present a prototype of a tiltable thermal chamber for the automatic analysis of MEMS IMU. The chamber, representing an evolution of the prototype proposed by the authors in [19], allows the automatic evaluation of the thermal behavior of the on-board accelerometer and additional sensors of MEMS IMU in the tilt measurement perspective. We tested the chamber analyzing the behavior of MEMS accelerometers of a number of MEMS IMU characterized by the same part number (i.e., same sensor board). Our analysis underlined a variable thermal behavior even for sensors belonging to the same model and the inconsistency of the theoretical behavior of these sensors as declared by the manufacturer (probably connected to the cabling system). Our analysis confirmed that for using low-cost MEMS IMU in tilt-based monitoring applications, where mid-to-high precision is required, a thermal analysis of each single sensor must be completed. This underlines the relevance of our development that is a considerable alternative to expensive commercial testing devices.

## 2. The Thermal Chamber Prototype

### 2.1. Concept and Hardware

**Figure 1:**
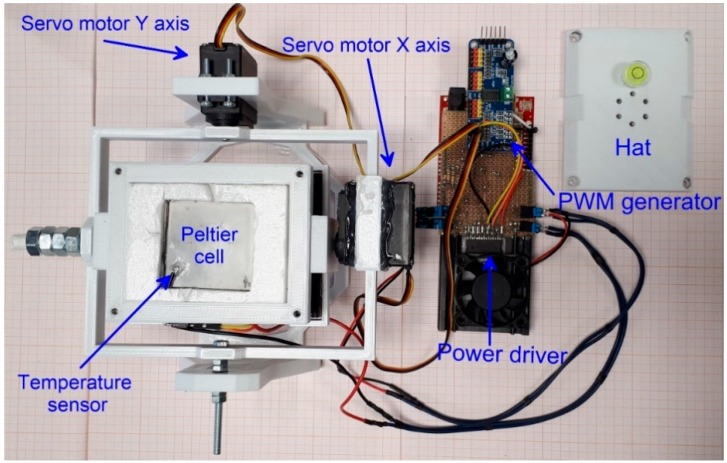
Top view of the developed thermal chamber prototype. Main components are labeled.

**Figure 2:**
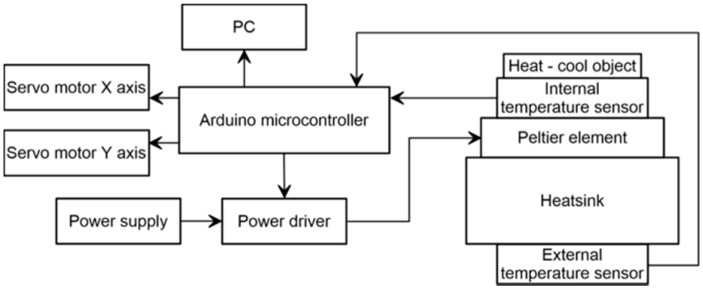
Diagram showing thermal chamber prototype components and functional connections.

The prototype of thermal chamber was developed to analyze the behavior of MEMS IMU onboard accelerometers in the tilt measurement perspective. The use of the onboard sensors for measuring inclination implies that their analysis needs to be extended in order to consider their behavior during inclination variation along a single or two axes. Thus, in system conceptualization we consider two technical aspects, a first related to the measurement of sensor response to temperature variation and a second related to the analysis of sensor response at different inclinations. For simulating temperature variation, we adopted the simplest way to create accelerated thermal stress consisting in supply power to a small Peltier element. To ensure tilting capabilities, we designed a frame composed of two parts, each representing a plane, that is tiltable along different axes (Figure 1). In this way, both single axis and biaxial sensors can be analyzed. On this basis, our system is formed by two elements. The first element is the thermal chamber that is composed of a (i) thermoelectric cooling and heating element (TEC), a (ii) power driver, and (iii) temperature sensors. The second element is the tilting device, composed of (i) a biaxial 3D-printed tilting frame and (ii) a couple of servomotors that control the inclination (Figure 1). System structure is designed to allow continuous tilting of both axes and is 3D-printed in order to maintain the overall cost of the prototype as low as possible obtaining reliable measurement and making sensor analysis at different inclination fully automatic. An Arduino^®^ UNO board equipped with a dedicated shield controls both the thermal chamber and the tilting device. A detailed scheme of the chamber is shown in Figure 2.

The thermoelectric element is a Peltier cell [20]. It is a two-side solid-state active pump allowing the heat transfer from a side to the other. The heat-flux direction between faces depends on the direction of the current that is regulated by the Peltier effect. The Peltier cell is composed of a series of P and N semiconductors arranged between two thin ceramic plates. The cooling and heating of each face occur as a consequence of current flow through the P and N semiconductors, and are regulated by the direction of the flux. Adsorbed heat of a face and dissipation through the other, $Q_{c}$ and $Q_{h}$ are, respectively [21]:

(1) $$Q_{c}=\alpha IT_{c}-\;\frac{1}{2}I^{2}R-k\Delta T$$

(2) $$Q_{h}=\alpha IT_{c}+\;\frac{1}{2}I^{2}R-k\Delta T$$

where $Q_{c}$ is the adsorbed heat, $Q_{h}$ is the dissipation heat, α is the Seebeck coefficient (*V/°K*), $T_{c}$ is the cold side temperature (°*K*), I is the input current (*A*), *k* is the thermal conductivity of TEC (*W*/*°K*), and Δ*T* is the temperature difference between two faces of TEC (°*K*).

For our prototype, we used a TEC1-12706 (http://www.hebeiltd.com.cn) Peltier element mounted on an aluminum heat sink to improve heat dissipation. It is characterized by a maximum power of 50 W, a maximum temperature difference between the faces of 66 °C, a maximum current of 6.4 A and a maximum voltage of 14.4 V. To modulate heat flux and consequently allow full-range temperature variation on a single face of the cell, we used a power drive based on a dual full-bridge L298N driver, which is an integrated 15-lead Multiwatt and Power SO20 monolithic package [22].

**Figure 3:**
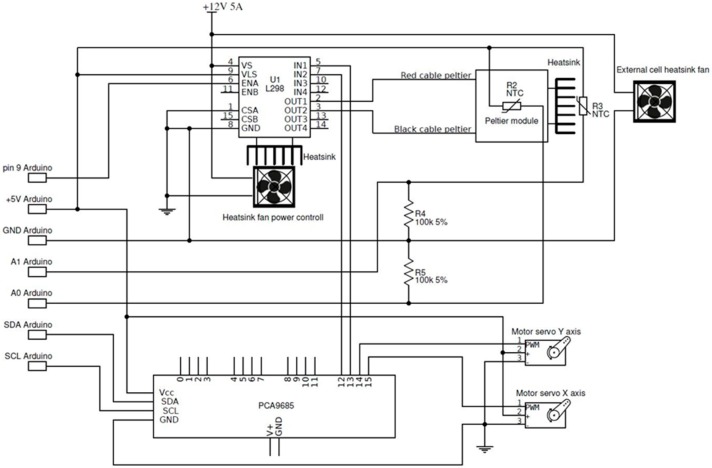
Electronic diagram of the thermal chamber and connections with the Arduino^®^ UNO board.

This device, using standard TTL logic, also allows to control the Peltier cell through a microcontroller. Figure 3 shows the wiring diagram of the thermal chamber and its connection with the Arduino^®^ board, in this case, an Arduino^®^ UNO board. Cell power is controlled using the Pulse Width Modulation [23,24] technique (PWM), which allows to turn the power on and off very quickly. Since the PWM resolution of Arduino board is only 8 bit (255 values), we chose a 12 bit (4095) external PWM generator board, based on PCA9685 chip, that should improve the behavior of the cell. This technique is used also to drive servomotors. Power modulation occurs through the ENA input pin of the L298N driver, while current flux direction is controlled by the IN1 and IN2 input pins as shown in Figure 3. These pins are connected to the digital outputs 9, 7, and 6 pins of the control board. Such board is an Aduino^®^ UNO low-cost and open source electronic board based on an ATmega 328P low-power CMOS 8-bit microcontroller. It has 14 digital I/O pins, 6 analog inputs, a 16 MHz quartz crystal, a USB connection, a power jack, an ICPS jeader and reset button (https://www.arduino.cc). 

Temperature control within the chamber is completed using two 100 KΩ CLW1064 thermistors, characterized by a tolerance of ±1%, installed with a reference resistor of 100 KΩ (tolerance of 5%). Each thermistor is installed on a face of the cell using a thermal compound. This is needed because the temperature of a side depends on the temperature of the opposite face, thus it is essential to control both sides of the cell. For temperature estimation of thermistors, we used the Steinhart–Hart equations:

(3) $$\frac{1}{T}=A+Bln\left(R_{th}\right)+C\;{\left[ln\left(R_{th}\right)\right]}^{3}$$

where *T* is the temperature (°*K*), *A*, *B* and *C* are Steinhart–Hart coefficients, $R_{th}$ is the resistance of thermistor (*Ω*). The thermistors were calibrated between −10 and 50 °C and obtained Steinhart–Hart coefficients were used for temperature estimation. Steinhart–Hart coefficients were calculated using a SRS Thermistor Calculator (https://www.thinksrs.com). We estimated temperature error by comparing thermistor measurements with a precision thermometer (accuracy ±0.05 °C) in laboratory controlled conditions. The RMSE calculated on the basis of 50 observations (between −10 and 45 °C) was ∼0.5 °C. The obtained temperature (*T*) is measured in °*K* and needs to be converted in °C.

The chamber, formed by TEC and heat sink, was installed on a servomotors-controlled tilting frame. It was designed using Blender software (https://www.blender.org/) and made of ABS (acrylonitrile butadiene styrene). It was printed using a 3D PRN LAB54 printer and an ABS filament of 1.75 mm in diameter (specific density 1488 kg m^3^). The filament was extruded at 210 °C with a velocity of 30 mm s^−1^. The structure, composed of multiple components, was designed to allow biaxial tilting of the chamber (design files stl are reported in the Supplemental). Tilt angle of X and Y axes can be setup independently, in a range of ±45 degrees for each axis. This is possible because of the mounting position of servomotors (Figure 1). Frame rotation is facilitated by two couples of ball bearings (0.85 mm of diameter) for each axis pivot. Axis rotation (i.e., titling) is driven by an analog Hitec HS-311 servomotor (torque of 3.0 kg/cm, supply voltage of 4.8 V). Each Servo motor is controlled by a PWM signal generated by a PCA9685 chip. The inner part of the tilting frame supports the heat sink over which the Peltier cell is mounted. To ensure an optimal heat transfer a thermal compound is used. On the opposite side of heat sink a fan responsible of heat dispersion is mounted. The TEC element is covered by a printed hat that, forming the upper part of the cell, and is thermally isolated using a 3 mm sheet of depron foam. The external connection between the tested IMU MEMS sensor and the acquisition board is completed using connection screws installed on the cover (i.e., cell hat). We designed several covers with different connection screw patterns for different sensor boards geometry. In addition, a bubble spirit level was installed at the top of the cover for visual check of cell tilting. 

### 2.2. Software and Workflow

**Figure 4:**
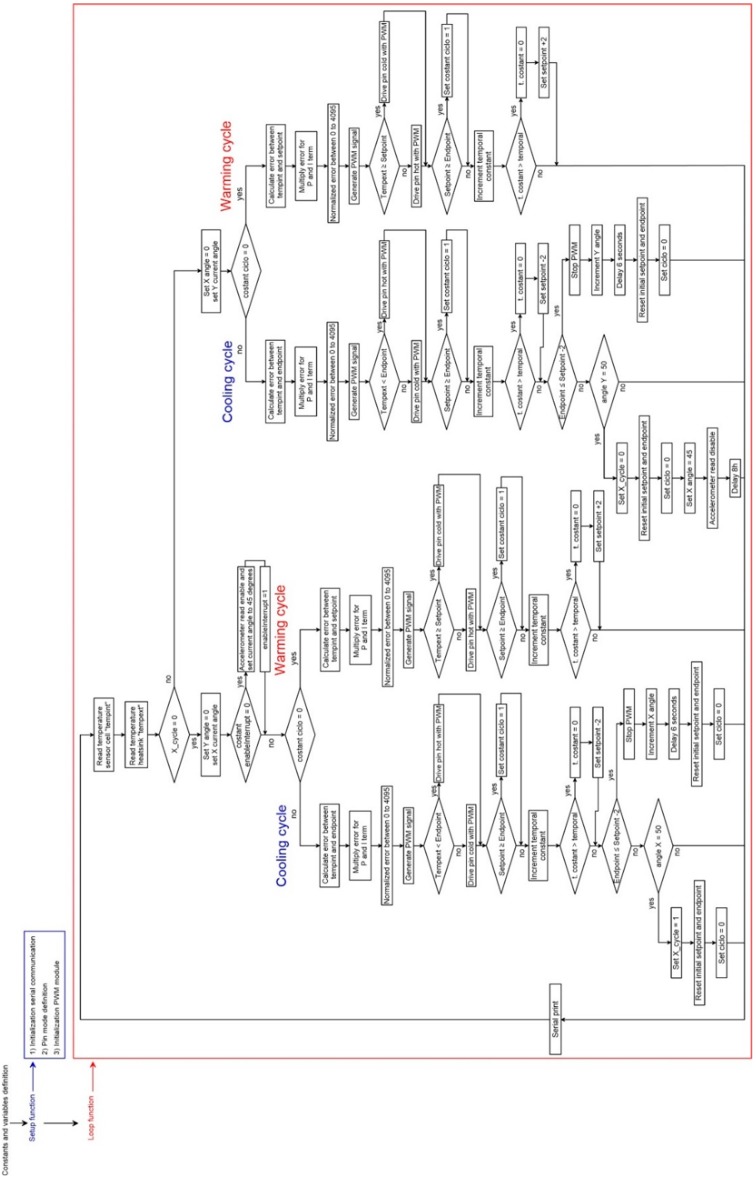
Flow chart showing the structure of the code used to control the thermal chamber.

**Figure 5:**
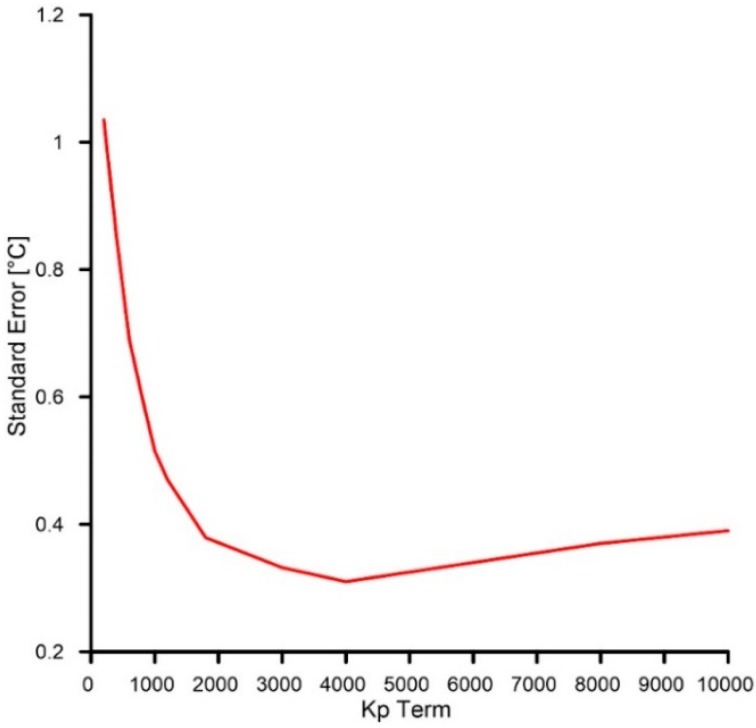
Graph showing *Kp* term and associated temperature error.

The logic of the code (reported in the Supplemental), developed for making the chamber work, is reported in Figure 4. The code, developed and compiled using the Arduino IDE environment, includes a two-way temperature cycle (warming and subsequent cooling cycles) and the automatic variation of inclination of both the X and Y axes. Its structure, derived following a sequential analytical approach (i.e., multiple conditions to be verified), accounts for a first phase of initialization and variable definition, a second phase of temperature sensor read that represent the chamber cold-start, and a last phase of testing. This last phase accounts primarily for X axis tilting and for each inclination step run a sequential warming and cooling testing cycles and subsequently it accounts for the Y axis titling and related thermal analysis. Both tilting and sequential thermal cycles are regulated though multiple “if” conditions based on continuous temperature readings (i.e., internal and external thermistors). The code is based on a simplification of the Proportional-Integral-Derivative algorithm (PID) [25] that, continuously modulating power supplied to the Peltier element through a Pulse Width Modulation, controls temperature variation. Especially, since the temperature of the reference face of the Peltier element is related to the temperature of the opposite face and the programmed temperature (to be reached), the temperature difference between the faces and the difference between the reference face and the programmed temperature are used as basic parameters to modulate power and to change power direction along the circuit. This algorithm has the form of a control loop feedback that, tending to minimize the difference between internal and programmed temperature, allows a dynamic response of the chamber thereby increasing accuracy and reliability of the system. In our case, it is integrated with a step function for controlling the tilt angle of each axis during thermal analysis. The PID algorithm [26] is used in its simplified form that considers only the P- and I-Terms for reducing the steady state error of the system as a function of the gain factor:

(4) $$P˗Term=K_{p}*e_{t}$$

(5) $$I˗Term=\int Ki*e_{t}dt$$

where *Kp* is the proportional gain, $K_{i}$ is the integral gain and $e_{t}$ is the error at time “*t*” (°C). As reported in Figure 4, the first step was the system setup (setup function), consisting of internalizing the serial communication, pin-mode, and PWM initialization. After this step, the system estimates the internal and external temperatures as the average of multiple measurements. Subsequently, on the basis of the estimated difference between the temperature of the reference face of the cell and the programmed temperature, the micro-controller calculates the power needed to shift up or down the temperature of the reference face. The power value is calculated multiplying the temperature difference (temperature error) for the *Kp* and *Ki*, which have been setup at 4000 and 0.05, respectively. For choosing the correct proportional gain *Kp*, we run an optimization experiment consisting of a series of thermal cycles with different *Kp* values. For each different *Kp* value, we estimated the standard error between the temperatures reached within the cell (with different *Kp* values) and the programmed temperatures during each complete cycle. This procedure allows us to identify the best *Kp* term related to the lowest standard error (see Figure 5).

The integral gain term *Ki* was chosen using a trial and error procedure. In our case, the *Ki* term was setup at 0.05 because higher values tend to destabilize the system. Since we used a 12-bit PWM power drive, the temperature difference needs to be converted in a value between 0 and 4095. After temperature stabilization for the predefined time interval, the temperature of the reference face is shifted up modulating the power.

**Figure 6:**
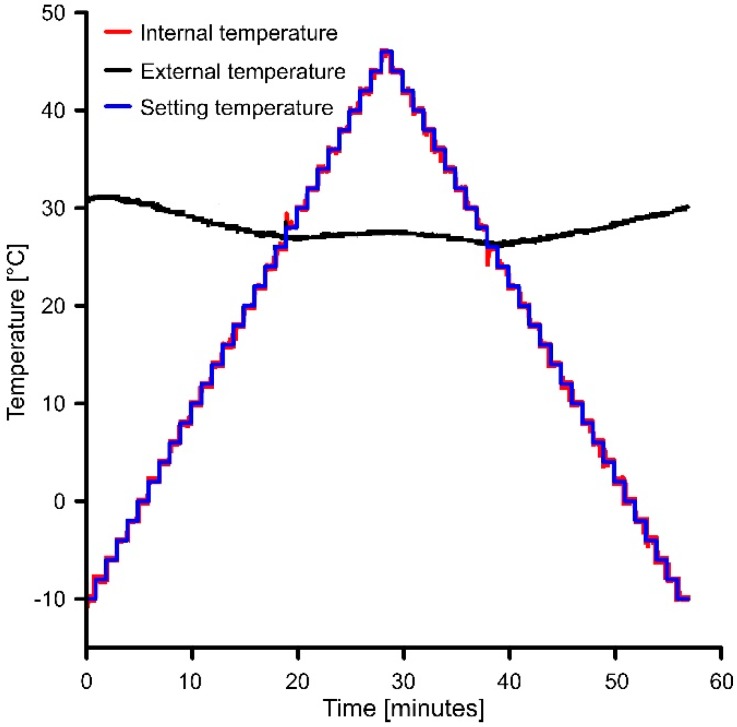
Example of temperature variation of the chamber during a complete thermal cycle (i.e., warming and cooling cycles).

**Figure 7:**
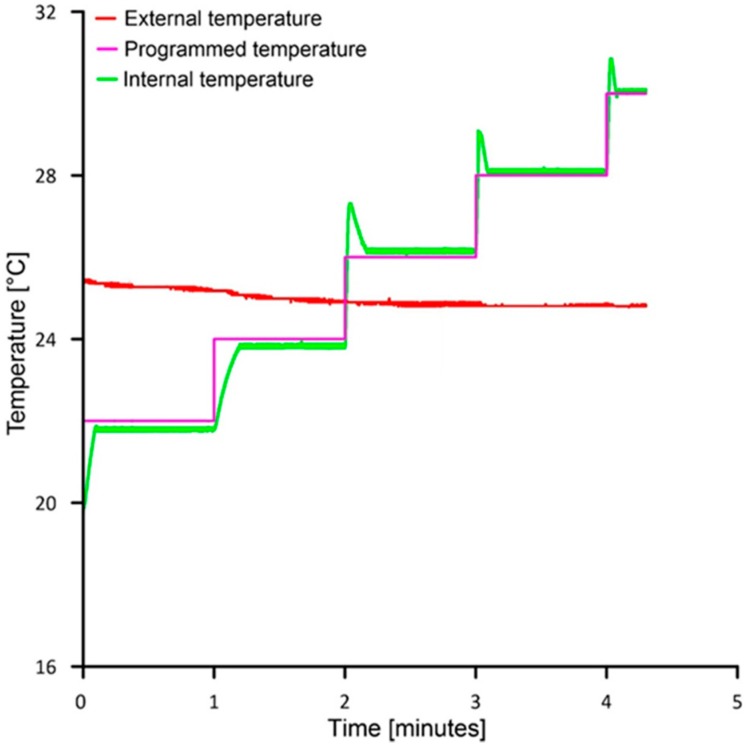
Example of temperature variation of the chamber during a warming cycle.

Figure 6 shows the data registered by the chamber (using the serial print function at the end of the code) during a complete thermal cycle. The black curve represents the variation of the external temperature as measured by the thermistor mounted on the heat sink. The blue stepped curve is the programmed temperature setup to variate with an increment of 2 °C and a duration of 1 min across a range between −10 and +45 °C. Figure 7 shows a further example of temperature variation during a 5-steps warming cycle. Graph of Figure 7 represents the behavior of the cell for a temperature variation from 20 to 30 °C. 

As shown in Figure 6 and Figure 7, in our configuration, the step function increases (or decreases during a cooling cycle) the temperature of 2 °C for each step of 1 min. Figure 7 suggests how the temperature variation of the reference face of the Peltier cell (green curve) is consistent with the programmed temperature variation (purple curve). Especially, if the internal temperature is lower than the external temperature, thermal stabilization time is about 15 s and the temperature is lower by 0.18 °C in comparison with that programmed. Conversely, when the internal temperature is higher than the external, stabilization time is shorter, about 6 s, and temperature is higher that the programmed of 0.15 °C. These temperature differences are directly related to PID algorithm parameters setting (i.e., *Kp* and *Ki*, see above). When a single warming measuring cycle ends, the controller checks if the programmed maximum temperature of the experiment is reached and, if so, it passes to the cooling cycle decreasing temperature step by step. Temperature decrease occurs through the inversion of the current direction within the TEC element. At the end of the cooling cycle, the cell is stopped for 1 min before to start a new temperature cycle. In our case, the cell is programmed so that the next thermal cycle is performed at a different inclination of the sensor along each axis at predefined step. In our case, the angular step was 5 degrees and the interval of variation was ±45 degrees for both X and Y axes. 

## 3. Application: Thermal Analysis of MEMS

### 3.1. Sensor and Interface

For our test of the thermal chamber prototype, we used LSM9DS0 MEMS IMU sensors board, also known as INemo^®^. Each sensor board is formed of three magnetometers, three axial accelerometers, three gyroscopes, and a temperature sensor. The IMU is equipped with an onboard 16-bit ADC, so that onboard sensors can be read through the I2C or SPI interfaces. Onboard accelerometers can be setup to work in a range of ±2, ±4, ±6, ±8, or ±16 g [27]. The sampling frequency is customizable in a range between 3.125 and 1600 Hz. Analog supply voltage is between 2.4 V and 3.6 V and the power consumption is ~7 mA. We chose this accelerometer because of its low cost, its resolution of 0.061 mg/LSB (Least Significant Bit), the measuring range of ±2 g, and the operating range of −40 to +85 °C. 

**Figure 8:**
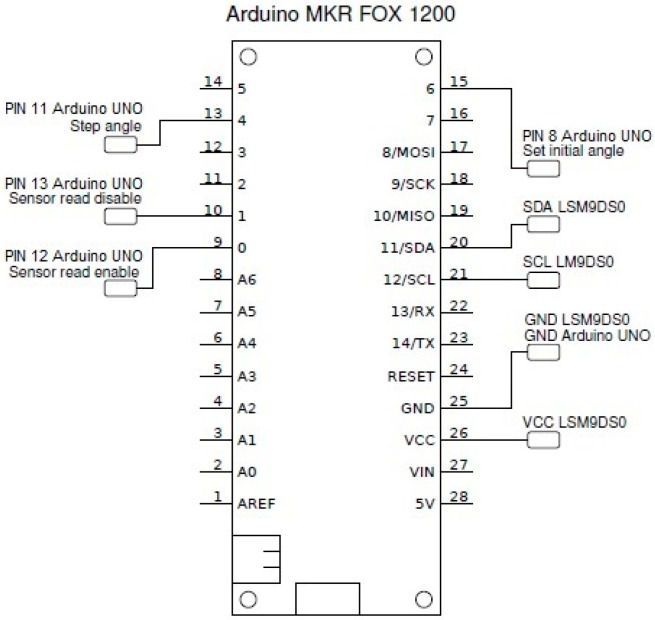
Wiring diagram of the LSM9DS0 Inertial Measurement Unit (IMU) with the Arduino^®^ MKR board.

For our experiment, the sensor board was connected to an Arduino^®^ MKR FOX 1200 board using the I2 C communication interface [28]. Figure 8 shows the wiring diagram of the sensor, which was powered up through the Arduino^®^ 3.3 Volt output pin (Vcc). 

**Figure 9:**
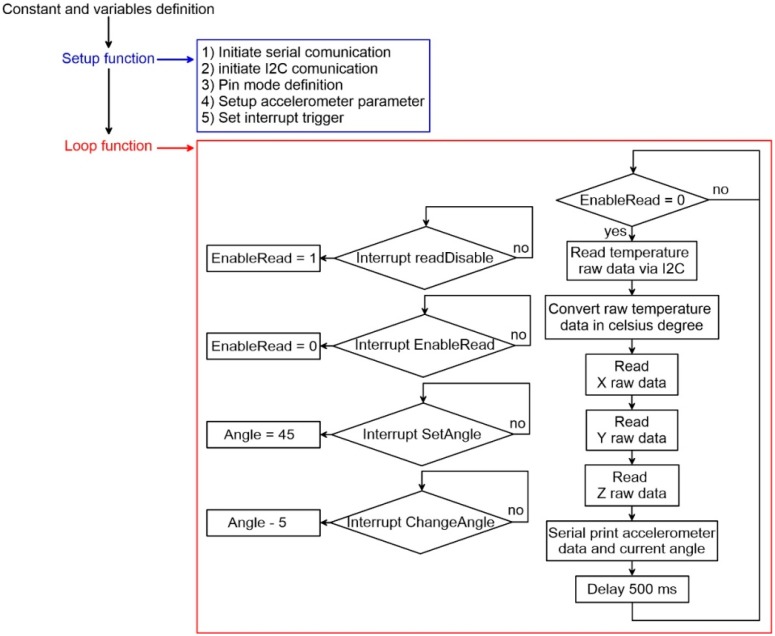
Flow chart showing the logic of the code used for reading the LSM9DS0 Micro Electro-Mechanical Systems (MEMS) sensor.

In order to synchronize sensor reading with thermal and angular data registered by the chamber, we connected the Arduino^®^ Uno board, driving the chamber, with the Arduino^®^ MKR, that is responsible for sensor reading. Board synchronization occurs through the interrupt pin of the Arduino^®^ MKR board. Figure 9 shows the logic of the code (reported in the Supplemental) used for reading, communicating and synchronizing with onboard accelerometers of the LSM9DS0 IMU. 

After parameters setup, raw data of each axis of accelerometer and temperature sensor are read through the I2C protocol (X, Y, Z and temperature). Raw temperature is also converted in °C considering the conversion factor of 8 LSB/°C. As indicated by the authors in [19], to solve a drawback consisting in a temperature measurement offset, we introduced an additional coefficient, estimated in 21.00 °C, that needs to be added to the converted value. This coefficient was estimated in laboratory-controlled conditions.

### 3.2. Thermal Analysis

**Figure 10:**
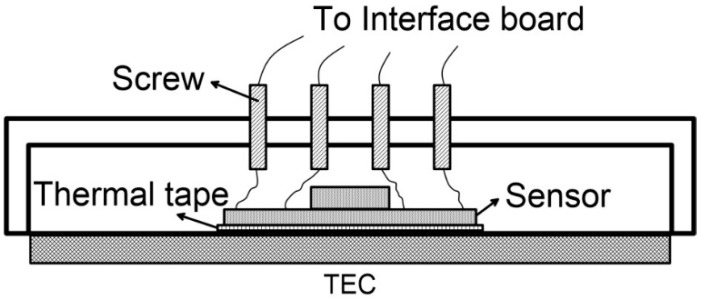
Schematic of sensor positioning within the cell.

Each sensor board was tested using the same procedure. As a first step the sensor was mounted on the TEC element surface ensuring that the X and Y axes of the sensors correspond to the X and Y axes of the chamber tilting device. To do so, we used a heat sink thermal tape (3M’s 8810) applied on the bottom side of the sensor DIE. This, in order to ensure the thermal continuity between the TEC element and the tested sensor as well as sensor position’s stability. The connection between the sensor mounted inside the cell and the reading external board was completed using 3.3 mm brass screws. Such screws are installed within the cell hat containing appropriate nuts (see scheme of Figure 10). 

After sensor mounting and connection, the chamber is positioned in an approximately horizontal position and then is powered up. Supply voltage is setup at 14 V (minimum 3 A needed). Once the cell is powered up, the first thermal calibration cycle began. By default, thermal cycles are completed with a temperature path from −10 to 45 to −10 °C (i.e., warming and cooling). Each cycle is completed with a different inclination of the X and Y axes in a range of −45 to 45° with a step of 5°. We arbitrary chose to start the thermal analysis variating the inclination of the X axis from an initial inclination of −45° and maintaining the Y axis in a horizontal position. After completing the analysis for each angular step of inclination of the X axis, the same procedure is completed for the Y axis. Overall, we made 38 complete thermal cycles, 19 of which completed variating the inclination of the X axis and 19 variating the Y axis. The whole procedure allowed us to acquire raw acceleration data for every single thermal cycle, at specific inclinations. Since both the sensor reading board and the chamber control board are connected to the pc thorough a USB interface, we used the CoolTerm (https://learn.sparkfun.com/tutorials/terminal-basics/coolterm-windows-mac-linux) software for reading/storing serial data sent from the boards. These raw data were used to calculate acceleration residuals as the difference between the measured acceleration at different temperatures and the reference acceleration at 25 °C. We chose this value as reference following the indication of the manufacturer. In this way, residual at 25 °C equals to zero [19].

### 3.3. Thermal Behavior of MEMS Accelerometer 

**Figure 11:**
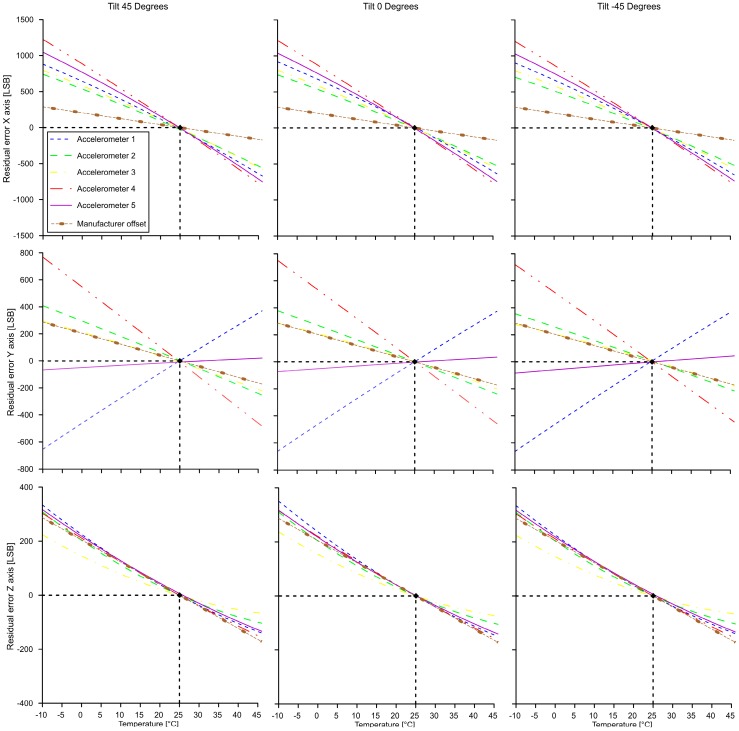
Results from the thermal analysis of MEMS IMU at different selected inclination.

Graphs of Figure 11 show examples of the results of our analysis of five MEMS IMU sensors in terms of acceleration residuals in a temperature range −10 to 45 °C. Graphs of the upper row are representative of the behavior of the IMU onboard accelerometer mounted along the X axis of the cell and tested at inclination of −45, 0 and +45°. Graphs of the central row are representative of the behavior of the IMU onboard accelerometer mounted along the Y axis of the cell and graphs of lower row are representative of the behavior of the IMU onboard accelerometer mounted along the Z axis. Table 1, Table 2 and Table 3 report the total drifting in the whole temperature range observed for each sensor and axis inclination expressed in LSB, as well as the mean error and the standard deviation. 

Our results indicate the existence of a variable thermal behavior of MEMS accelerometers installed on the IMU MEMS boards, also produced with the same standards. The graphs in Figure 11 (central row) demonstrate that this variability is more accentuated for the Y axis. In addition, our data underline the different thermal behavior of axial accelerometers mounted on a single IMU board. The X axis showed the highest thermal drift while the Z axis had lowest. This variability in sensor behavior might be connected to the sensor design, constitutive materials and cabling system and is related to the physical deformation of the sensor [29,30,31]. In this case, considering that the MEMS IMU boards are produced with the same standards, the observed different behavior might result from the productive process. Micrometric differences in MEMS production might have repercussions on axial accelerometer thermal behavior. In these conditions, general manufacturer indications about MEMS thermal drift (in our case ±0.5 mg/°C) might not be sufficient for an aggressive compensation of the sensor and its subsequent use in medium to high accuracy monitoring applications. Thus, specific thermal analysis is required. 

## 4. Conclusions

In this work, we presented a complete measuring system prototype for the thermal characterization of low-cost MEMS IMU sensors in tilt measurement perspective. The development of this system is configured as a response to the need of compensating thermal drifting that inhibit the use of low-cost sensor board for environmental and structural monitoring applications based on inclination measures. The system, as described in this paper, is formed by a 3D printed biaxial tiltable frame, a Peltier-element based chamber, and a control and power system. It is controlled by a micro-controller, in our case an Arduino^®^, and a self-made board that include a TEC driver and servo-motors driver. Peculiarities of our thermal chamber are low cost (about 250 EUR), open-source, simple reproducibility and easy retrieval of the electronic components. 

Our test of the chamber prototype, consisting in the thermal analysis of multiple IMU MEMS board of the same model, indicates a variable thermal behavior of each axial accelerometer of a single board as well as of accelerometers materializing the same axis of multiple boards. This variability underlined the need for a complete thermal analysis of low-cost MEMS-technology-based measuring sensors that is the basis for the development of a dedicated compensation strategy. This is a key aspect in making low-cost sensors suitable in medium-to-high precision monitoring applications where reliability and reproducibility are needed. In addition, the very low cost of the chamber makes sensors thermal analysis accessible to a wide range of users that have no resources for expensive commercial products.

## Figures and Tables

**Table 1:** Total drifting of the accelerometer materializing the X axis each IMU MEMS board in a −10° to +45° temperature range experiment (expressed in Least Significant Bit, LSB) for the three sample inclinations.

Accelerometer	Total Error X Axis 45	Total Error X Axis 0	Total Error X Axis −45
1	1553	1553	1553
2	1300	1262	1223
3	1373	1367	1362
4	2037	2014	1991
5	1797	1781	1764
Mean	1612	1595.4	1578.6
Standard Deviation (**σ**)	273	273.8	275.1

**Table 2:** Total drifting of the accelerometer materializing the Y axis each IMU MEMS board in a −10° to +45° temperature range experiment (expressed in LSB) for the three sample inclinations.

Accelerometer	Total Error Y Axis 45	Total Error Y Axis 0	Total Error Y Axis −45
1	1024	1037	1048
2	662	617	571
3	515	485	457
4	1252	1226	1165
5	88	108	128
Mean	708.2	694.6	673.8
Standard Deviation (**σ**)	404.8	398.4	383.8

**Table 3:** Total drifting of the accelerometer materializing the Z axis each IMU MEMS board in a −10° to +45° temperature range experiment (expressed in LSB) for the three sample inclinations.

Accelerometer	Total Error Z Axis 45	Total Error Z Axis 0	Total Error Z Axis −45
1	500	474	500
2	415	412	415
3	348	291	348
4	479	460	479
5	458	451	458
Mean	440	417.6	440
Standard Deviation (**σ**)	53.9	66.5	53.9

## Supplementary Materials

The following are available online at https://www.mdpi.com/1424-8220/19/12/2705/s1.
